# Molecular Interaction Characterization Strategies for the Development of New Biotherapeutic Antibody Modalities

**DOI:** 10.3390/antib9020007

**Published:** 2020-03-25

**Authors:** Xiangdan Wang, Minh Michael Phan, Ji Li, Herman Gill, Simon Williams, Nidhi Gupta, Valerie Quarmby, Jihong Yang

**Affiliations:** 1 Department of BioAnalytical Sciences, Genentech, South San Francisco, CA 94080, USA; phanm1@gene.com (M.M.P.); quarmby@gene.com (V.Q.); 2 Department of Translational Oncology, Genentech, South San Francisco, CA 94080, USA; jli@gene.com; 3 Department of Biomedical Imaging, Genentech, South San Francisco, CA 94080, USA; hermang@gene.com (H.G.); williams.simon@gene.com (S.W.); 4 Department of Immunology, Genentech, South San Francisco, CA 94080, USA; Gupta.nidhi@gene.com

**Keywords:** affinity, molecular interaction characterization, new biotherapeutic antibody modality

## Abstract

The characterization of target binding interactions is critical at each stage of antibody therapeutic development. During early development, it is important to design fit-for-purpose in vitro molecular interaction characterization (MIC) assays that accurately determine the binding kinetics and the affinity of therapeutic antibodies for their targets. Such information enables PK/PD (pharmacokinetics/pharmacodynamics) modeling, estimation of dosing regimens, and assessment of potency. While binding kinetics and affinities seem to be readily obtained, there is little discussion in the literature on how the information should be generated and used in a systematic manner along with other approaches to enable key drug development decisions. The introduction of new antibody modalities poses unique challenges to the development of MIC assays and further increases the need to discuss the impact of developing context-appropriate MIC assays to enable key decision making for these programs. In this paper, we discuss for the first time the challenges encountered when developing MIC assays supporting new antibody modalities. Additionally, through the presentation of several real case studies, we provide strategies to overcome these challenges to enable investigational new drug (IND) filings.

## 1. Introduction

Since the first approved clinical therapeutic in 1985 [1], antibodies have become a highly successful class of biotherapeutics with over 80 products approved by the Food and Drug Administration (FDA) [2] and hundreds more currently in clinical development for a variety of diseases. Molecular interaction characterization (MIC) of therapeutic antibody binding to target is critical to every stage of the therapeutic development process, with binding affinity data, in particular, considered essential for lead identification and characterization at the early discovery and development stages. During early development, it is important to design fit-for-purpose MIC assays that accurately determine the binding affinity of therapeutic antibodies to their targets for PK/PD (pharmacokinetics/pharmacodynamics) modeling, estimation of dosing regimen and assessment of efficacy. Despite the long history of biotherapeutic development, there is little discussion in the literature on how MIC data should be generated and used to enable key drug development decisions. As there are no clear regulatory guidelines in this area, each individual laboratory tends to apply its own practices. This has sometimes resulted in dramatically different information [3,4] which not only makes it difficult to compare MIC data between laboratories, but could also add confusion and delays in the drug development.

Early clinical therapeutic products were dominated by chimeric [5,6], humanized [7,8,9], and fully human monoclonal antibodies [10,11] with conventional properties. More recently, new modalities with more complex molecular structures and properties have been introduced such as antibody drug conjugates (ADCs) [12,13], PEGylated antibody fragments [14,15], immuno-positron emission tomography (PET) imaging antibodies [16], and bi-specific antibodies [17,18]. The more complex structures and properties of these new modalities provide various advantages over conventional antibodies including selective delivery of cytotoxic drugs to improve anti-cancer effect while minimizing systemic toxicity [19], improved pharmacological properties [14], enhanced efficacy through targeting multiple pathways or engagement of T cells [20], and non-invasive measurement of the biodistribution of antibodies [16]. Meanwhile, they have posed unique challenges to the development of MIC assays, and further increased the needs to discuss how MIC data should be generated to support the development of these specific programs in the pharmaceutical community.

MIC assays that are commonly used to determine the binding affinity of therapeutics to their targets can be classified into two categories: cell-based equilibrium assays and non-cell-based assays that rely on purified or recombinant target proteins. Cell-based equilibrium assays are well-established for the determination of the binding affinity of a ligand to cell-expressed target proteins [21]. The majority of these assays rely on incubating a radioisotopically or fluorescently labeled ligand on cells expressing the receptor of interest and detecting the fraction of bound ligand after equilibrium is reached. There are two major types of equilibrium assays: saturation experiments measure binding of various concentrations of a labeled ligand; competition experiments measure binding of a fixed concentration of the labeled ligand mixed with various concentrations of unlabeled competitor. A binding isotherm is then generated by plotting the amount of bound ligand as a function of free concentration of ligand. From the binding isotherm, the binding affinity, expressed as an equilibrium dissociation constant (K_D_), can be derived; this is the concentration of the ligand required to occupy or compete 50% of the binding sites. 

Technologies that are commonly used to develop non-cell-based assays for affinity measurement include surface plasmon resonance (SPR) (i.e., Biacore [22,23,24] and ProteOn [25]), biolayer interferometry (Octet) [26,27], analytical ultracentrifugation [28,29], isothermal titration calorimetry [30,31], and KinExa [32,33]. Multiple studies including a benchmark study have been conducted to compare binding affinities derived using some of these technologies [34,35]. Among them SPR is the most commonly used, and has been considered the gold standard in the industry for measuring kinetics and affinity of biomolecular interactions during early or advanced stages of biotherapeutic development. In an SPR assay, recombinant or purified protein molecules are immobilized or captured on a gold sensor surface and a potential binding partner in solution is injected over the surface to allow the interaction to take place. The interaction profile is recorded in real time as a sensorgram, enabling measurement of the affinity derived from kinetic parameters, as well as from the equilibrium state analysis. 

MIC assays in each category have strengths and weaknesses. Cell-based equilibrium assays are often considered, since cell-expressed receptors are generally deemed more biologically relevant than the recombinantly expressed proteins. However, depending on the availability of cell lines, receptor expression levels and the biophysical properties of the evaluated therapeutics, it is not always feasible to develop a reliable cell-based assay for affinity measurement. Cell-based assays are often run at 4 °C or 25 °C to minimize receptor internalization. As affinity can be highly temperature dependent [36], K_D_ values determined at these temperatures may not be representative of those measured at physiologically relevant temperature, 37 °C. Furthermore, it can be challenging to measure interactions with very high binding affinities (e.g., K_D_ value in pM range) using cell-based equilibrium assays as it may take extended periods of time to reach equilibrium and the cell viability can be severely compromised. SPR is capable of measuring affinity in a wide range (K_D_ value from mM to pM), and is thus suitable for studying most biomolecular interactions. Additionally, it provides kinetic information that can be very useful for understanding the biological processes behind an interaction [37,38]. As SPR heavily relies on recombinant or purified proteins, its application is limited for proteins that are challenging to produce due to aggregation or poor stability such as some membrane proteins. Additionally, in the process of recombinant protein production, protein misfolding and post-translational modifications may occur, providing potentially biased binding properties compared with proteins naturally expressed by cells. Furthermore, considerations should be taken when designing an SPR assay and interpreting the results since the immobilization or capture steps could modify the binding properties of the ligand protein [3,4].

Compared to conventional mAbs, MIC assay strategies for new antibody modalities are more complex. The complex molecular structure and properties of these molecules create additional challenges for characterizing the binding of these molecules to their targets. For many bispecific antibodies, both of the therapeutic targets are expressed on cells, which might have low expression levels and also hard to purify as recombinant proteins. Often, a combination of multiple MIC assays is required to fill in the gaps and provide confidence in the data. For conjugated antibody modalities such as ADCs, PEGylated antibody fragments, and immuno-PET imaging agents, characterization of the target binding of both unconjugated and conjugated forms is needed to assess the extent to which conjugation impacts binding. The additional characterization not only increases the number of interaction pairs to be evaluated, but could also pose challenges to the development of MIC assays, since the conjugation process can change molecular properties such as increased hydrophobicity of ADCs [39,40]. Furthermore, the conjugation reaction results in a heterogeneous mixture of antibodies with different conjugation ratios. The heterogeneity of the molecules could make the data analysis challenging in MIC assays where homogeneous proteins with high purity are usually required. As each molecule is unique and each program has different needs, it is important to design a fit-for-purpose MIC assay strategy for each program. In this paper, we share four case studies and describe the MIC assay strategies that were applied for several new antibody-based modalities during early development for the purpose of enabling investigational new drug (IND) filings. 

## 2. Case Study 1—MIC Strategy to Characterize Anti-MSLN ADC Binding to Targets

The anti-mesothelin (MSLN) ADC consists of three moieties: the antibody that recognizes MSLN, a protein that is over-expressed on cell surfaces in several types of cancer [41], monomethyl auristatin E (MMAE), a potent cytotoxic agent that can depolymerize microtubules and prevent cell division, and a protease-labile linker, maleimidocaproyl-valine-citrulline-*p*-aminobenzyloxycarbonyl (MC-VC-PAB), that joins the antibody and MMAE together. The mechanism of action of anti-MSLN ADC is similar to that of other ADCs: following target-specific binding of the ADC on the cancer cell surface, the ADC can be taken into the cell and the cytotoxic drug is released, resulting in disruption of the microtubule network, inhibition of cell division and growth, and tumor cell death [42,43,44]. The anti-MSLN ADC has the potential for the treatment of mesothelin-positive ovarian and pancreatic tumors, and its clinical effects have been reported [45,46].

There are several unique aspects of this program. The clinical lead antibody only binds to human MSLN, so a surrogate antibody was produced in order to assess its safety in the species used for toxicity studies, cynomolgus monkey and rat. Additionally, it was found that the glycosylation level of the MSLN extracellular domain (ECD) affected the surrogate antibody binding to human MSLN in vitro, but had no impact on the lead antibody binding to the human target. The goal of the IND-enabling MIC study was to evaluate binding affinities of the lead and surrogate antibodies to their targets. In addition, it was important to assess the impact of MMAE conjugation on target binding of the anti-MSLN antibodies. 

As the binding of the surrogate antibody to human MSLN is affected by the glycosylation level of MSLN ECD proteins, it is expected that the binding of the surrogate antibody to the cynomolgus monkey and rat MSLN would also be affected. For this reason, cell-based radiolabeled equilibrium assays were chosen to evaluate the MSLN antibodies binding to their targets. Table 1 summarizes binding affinity data determined by radiolabeled equilibrium assays using different cell lines. The MSLN lead and surrogate antibodies bound to human, cynomolgus monkey, and rat MSLN-expressing cell lines with affinities of 0.2–1 nM [47], 1.3–6.4 nM, and 2.7–7.3 nM, respectively. The radiolabeled equilibrium assays, however, were only able to determine affinities of the unconjugated antibodies binding to MSLN proteins expressed on different cell lines. For ADC antibodies, the background signals in the assay were too high, precluding a determination of reliable K_D_ values. The high assay background is likely caused by significant non-specific binding of the ADC antibodies to the cell surface, since ADC antibodies overall are more hydrophobic than unconjugated antibodies [39,40], a phenomenon that has been observed in several other ADC projects (internal unpublished data). Therefore, an alternative SPR binding assay was developed in order to characterize ADC antibodies binding to their targets. This also allowed comparisons of binding kinetics and affinities between the unconjugated and conjugated MSLN antibodies. 

To circumvent the impact of glycosylation on the binding, MSLN ECD proteins expressed in *E. coli* were first tested in the SPR assay as these proteins are non-glycosylated. The MSLN ECD proteins expressed *in E. coli* were provided in acidic buffers (pH 3.0) to improve solubility, and were in denatured and reduced forms. Although the proteins were successfully used in an ELISA-based PK assay as a coat reagent to capture MSLN ADC molecules, it is necessary to evaluate whether the MSLN ECD proteins exhibit binding properties suitable for SPR analysis. To address the question, the proteins were immobilized on a sensor chip and MSLN antibodies were injected as analytes. As shown in Supplementary Figure S1A, the experimentally determined maximum response (R_max_) (E) values for all three tested target ECD proteins were found to be much lower than the theoretically calculated R_max_ (T), and the ratios of R_max_ (E) to R_max_ (T) for all three targets were less than 20%, much lower than that generally recommended (over 70–80%) by the manufacturer. As lysine is not involved in the key binding epitope of the MSLN antibodies, the unacceptably low binding responses were likely caused by the impaired binding properties of the purified ECD proteins instead of being introduced by the amine coupling immobilization reaction, although modification of lysine residues could lead to steric hindrance and affect the protein binding indirectly. To investigate the cause of the low binding responses in the SPR assay, recombinant human and cynomolgus monkey MSLN ECD expressed in Chinese Hamster Ovary (CHO) cells were produced, and the fractions with the lowest levels of glycosylation were collected and tested in the assay. As shown in Supplementary Figure S1B, the ECD proteins expressed in CHO cells had much higher binding responses. The ratios of R_max_ (E) and R_max_ (T) for human and cynomolgus monkey MSLN ECD were 85% and 78%, respectively. This result suggested that it is unlikely that the amine coupling procedure modified the binding properties of the MSLN ECD proteins. Moreover, it showed that the quality of the *E. coli*-produced MSLN ECD proteins were not suitable for use in the MIC assay.

SDS-PAGE characterization of the ECD proteins expressed in CHO cells showed that the fraction with the lowest amount of glycosylation was still very heterogeneous, with multiple bands on gels (data not shown). To minimize the impact of heterogeneity on data analysis, the MSLN ECD proteins were immobilized on the sensor chip, and the conjugated antibodies were injected as analyte in the flow. As each MSLN ECD bears one binding site for the MSLN antibody, it is possible that one antibody could bind to two immobilized MSLN ECD molecules despite the effort of immobilizing MSLN ECD at a low density on the sensor chip. For this reason, both monovalent and bivalent analyte binding models were evaluated for describing the binding interactions. A much greater discrepancy was observed between the experimental data and the fitted curves using a 1:1 monovalent binding model than was obtained from the bivalent binding model in all cases (data not shown), suggesting that the 1:1 binding model did not adequately describe the interactions between MSLN antibodies and MSLN ECD. 

As shown in Table 2, the averaged K_D1_ values determined using a bivalent binding model for the parental and ADC-conjugated lead MSLN antibodies binding to recombinant human MSLN ECD were 1.4 and 2.3 nM, respectively. The values for the parental and ADC-conjugated surrogate antibodies binding to recombinant cynomolgus monkey MSLN ECD were 7.9 and 11.9 nM, respectively. These affinity data were similar to those derived from the radiolabeled equilibrium assays. Under the experimental conditions described herein, the binding affinities and the kinetics of the conjugated MSLN antibodies were not significantly different than those observed with the unconjugated antibodies. 

In this case study, both the cell-based radiolabeled equilibrium assay and the SPR assays were used to cross-validate and provide confidence in measured affinities. This strategy enabled a successful IND filing by confirming the appropriate dose selection in the cynomolgus monkey toxicity study. It also highlighted the importance of producing and characterizing each of the reagents employed to facilitate the development of an MIC assay. 

## 3. Case Study 2—MIC Strategy to Characterize T-Cell-Dependent Bispecific Anti-FcRH5/CD3 Binding to the Targets

1G7 is a humanized bispecific antibody directed against the ECDs of the fragment crystallizable receptor-like 5 (FcRH5) and the epsilon chain of cluster of differentiation 3 (CD3ε) antigens. FcRH5 is an Ig superfamily transmembrane protein; its expression is highly restricted to the B-cell lineage, including plasma cells and all multiple myeloma (MM) tumor cells [48]. CD3 is the co-receptor of T-cell receptor (TCR), and the TCR/CD3 complex is predominantly expressed on T-cells, including cytotoxic T cells (CD8+) and T helper cells (CD4+). The CD3 molecule contains four distinct transmembrane chains, and contains a gamma (γ) chain, a delta (δ) chain, two epsilon (ε), and two zeta (ζ) chains. Ligand-binding of TCR/CD3 complex with the major histocompatibility complex (MHC): peptide or MHC-independent binding of TCR/CD3 with CD3 antibody can induce T-cell activation. Engagement of both arms of the FcRH5/CD3 bispecific antibody results in T-cell-directed cell killing of FcRH5 expressing malignant cells for the treatment of MM [48].

In this study, it was necessary to characterize the binding affinities of 1G7 to both FcRH5 and CD3 from both human and cynomolgus monkey to verify receptor occupancy (RO) of a proposed first-in-human dose and to justify the species selection for toxicity studies. Preliminary binding data generated at the early discovery stage of the program suggested that 1G7 binding to both targets is temperature dependent. As the binding affinity value is important to verify RO in the first human study, it is important to derive accurate affinity data at physiologically relevant temperature (37 °C). On the other hand, like many other transmembrane proteins, recombinant FcRH5 and CD3ε ECD proteins are very difficult to express and purify. Despite all efforts, high quality recombinant cynomolgus monkey ECD proteins for both targets were unavailable. Considering these challenges, the MIC assay strategy for this program was to combine results from both cell-based assays and the SPR assay to enhance data confidence. 

**Figure 1:**
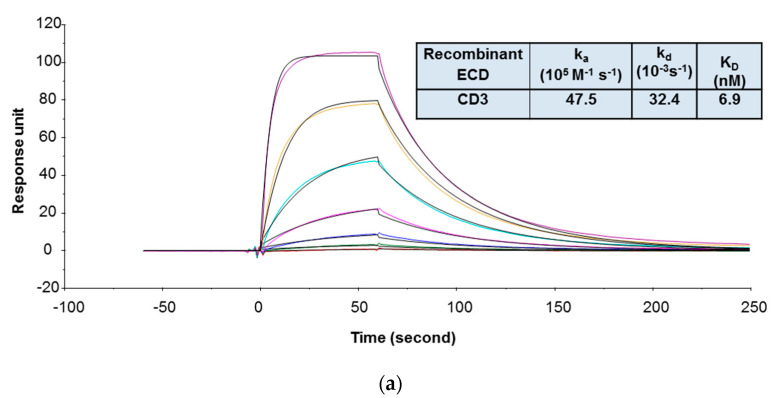

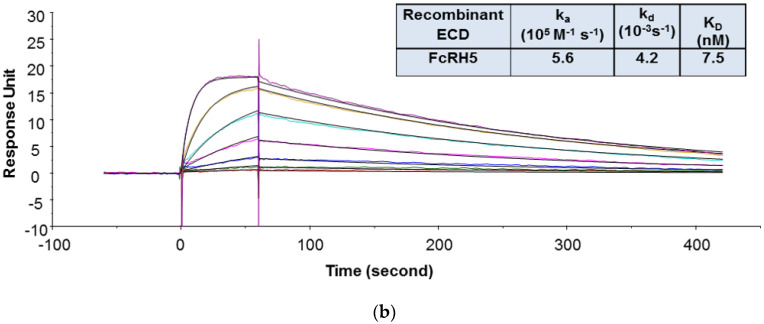
Representative sensorgrams of concentration-dependent binding of 1G7 to recombinant human CD3 ECD (**a**) and FcRH5 ECD (Domain 9) (**b**) at 37 °C. Note: Black solid lines are fitted curves using a 1:1 binding model, and colored lines are the actual data. 1G7 concentrations (from bottom to top) in 1A are 0.08, 0.23, 0.69, 2.06, 6.17, 18.52, and 55.56 nM; recombinant human FcRH5 Domain 9 ECD concentrations (from bottom to top) in 1B are 0.93, 2.3, 5.8, 14.5, 36.2, 90.4, and 226 nM. The sensorgrams were generated after in-line reference cell correction followed by buffer sample subtraction. The kinetics and affinity data in the tables are averaged from three independent experimental SPR runs.

In the SPR assay, the binding kinetics and affinity of 1G7 to recombinant human CD3 was determined using a format in which the recombinant human CD3 containing covalently linked extracellular domains of ε and γ subunits (CD3εγ ECD) was immobilized on the sensor chip and 1G7 was injected in the flow as analyte. The recombinant human CD3εγ ECD was prone to aggregation, and this format was found less sensitive to aggregation of human CD3εγ ECD. During the purification process, only the monomeric fraction of the recombinant human CD3εγ ECD was collected and stored. However, prior to the SPR study, size exclusion chromatography with multi-angle static light scattering (SEC-MALS) analysis revealed that this material still contained a high amount of aggregates (~30%). Although aggregates were removed again and the protein was reformulated in a buffer that minimizes aggregate formation, it was not clear whether aggregates would form when the recombinant human CD3εγ ECD was used in the SPR binding study. As shown in Figure 1a, by using this format, the K_D_ value of 1G7 binding to the recombinant human CD3εγ ECD was 6.9 nM. This format, however, could not be used to determine 1G7 binding to recombinant FcRH5 ECD because there was no detectable binding response of 1G7 to immobilized recombinant human FcRH5 ECD, indicating that the ECD protein lost binding activity after being immobilized on the sensor chip. For this reason, the binding kinetics and affinities of 1G7 to the recombinant human FcRH5 ECD was evaluated with an alternative format in which 1G7 was indirectly captured on the sensor chip via a monoclonal antibody against the Fc domain of human IgGs, and the ECD proteins were injected as analytes. An indirect capture format is often used in SPR analysis to evaluate protein–protein interactions [3,49]. The indirect format offers several advantages for kinetic analysis: (1) Ligand protein is not chemically modified; (2) ligand molecules can be oriented in a more uniform manner on the surface; (3) ligand binding properties can be preserved without deleterious effects of regeneration conditions; and (4) interference from ligand impurities can be minimized. As shown in Figure 1b, the K_D_ value of 1G7 to the recombinant human FcRH5 ECD was 7.5 nM.

Table 3 summarizes the binding affinities of 1G7 to FcRH5- and CD3-expressing cells determined by equilibrium binding assays. The averaged K_D_ values of 1G7 binding to human and cynomolgus monkey FcRH5 expressed in SV40 transformed fibroblasts (SVT2) cell lines were 3.1 nM and 8.2 nM, respectively [50]. The averaged K_D_ value of 1G7 binding to human CD3ε expressed on the Jurkat T-cell line was 2.6 nM. Binding affinity of 1G7 to cynomolgus monkey CD3ε was not determined when the assay was needed. In order to bridge the 1G7 binding results between human and cynomolgus monkey CD3ε, a flow cytometry assay was developed to compare binding responses of 1G7 to human and cynomolgus monkey CD3ε expressed on CD8-positive T cells sorted from peripheral blood mononuclear cells (PBMCs). As shown in Table 3, the concentrations of 1G7 at half maximal response determined from the flow cytometric assays for human and cynomolgus monkey CD3ε were 4.2 nM and 2.2 nM, respectively. Those results indicated comparable binding of 1G7 to human and cynomolgus monkey CD3ε, and thus justified the toxicology species selection. 

In this case study, SPR and cell-based assays including flow cytometric and radiolabeled equilibrium assays were combined to determine binding affinity of FcRH5/CD3 antibody binding to its bispecific targets. The strategy addressed the unique challenges of the program and mitigated the potential risk of delayed timelines caused by production of challenging proteins and assay development. This study highlighted the need to characterize reagents right before they are used in the MIC assays.

## 4. Study 3—MIC Strategy to Characterize PEGylated Anti-FD Binding to Targets 

**Figure 2:**
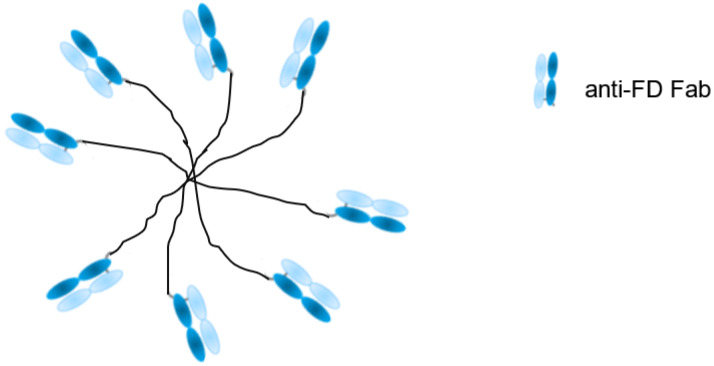
Schematic structure of CCFD9260S: eight anti-factor D (FD) Fabs conjugated on PEG.

Factor D (FD) is a highly specific chymotrypsin-like serine protease that is a rate-limiting enzyme of the complement alternative pathway. The substrate for FD is another alternative pathway serine protease, factor B (FB). Following cleavage by FD, FB is converted into the proteolytically active factor Bb, whereupon it initiates the alternative complement pathway [51]. Increased activation of the alternative complement pathway has been found in drusen, cytotoxic complement-containing deposits present on Bruch’s membrane. They are associated with the development of age-related macular degeneration (AMD) [52]. The lead molecule, CCFD9260S, consists of anti-FD Fab fragments that are covalently conjugated to a polymeric polyethylene glycol (PEG) molecule (Figure 2). This complex has therapeutic potential for dry AMD patients with geographic atrophy. PEGylation is a well-established technology, and has been widely used as a strategy to extend the half-life of Fabs [53,54]. 

**Figure 3:**
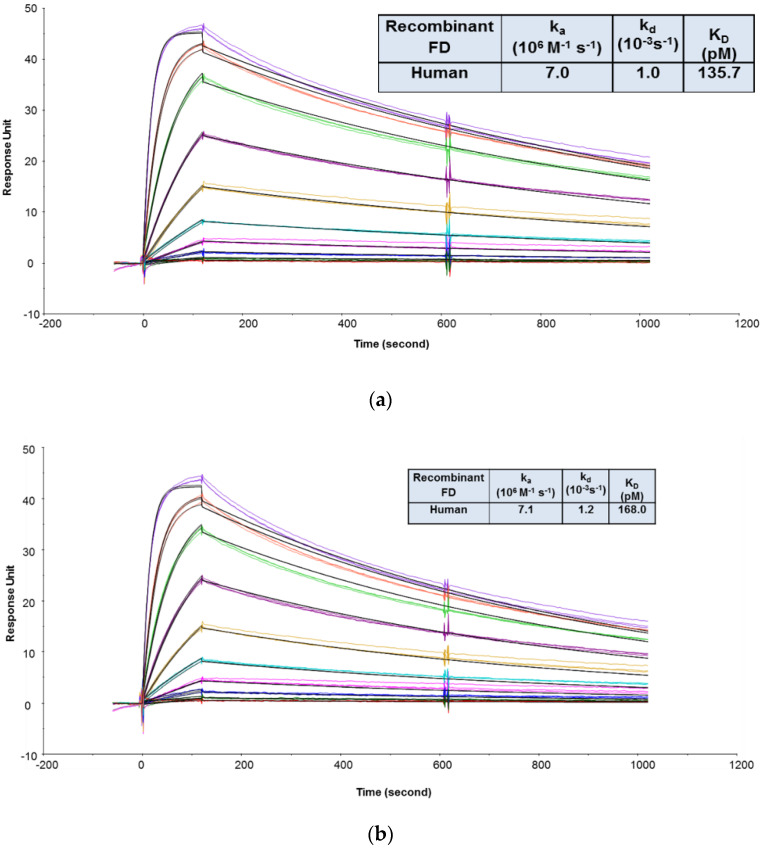

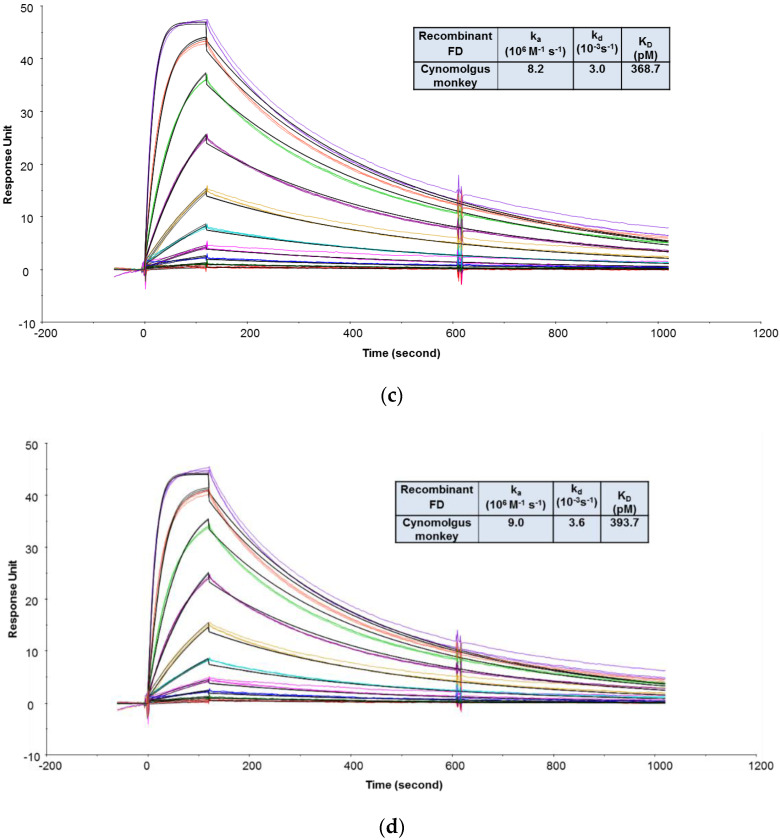
Representative sensorgrams of concentration-dependent binding of recombinant human FD to CCFD9260S (**a**) and anti-FD Fab (**b**), and recombinant cynomolgus monkey FD to CCFD9260S (**c**) and anti-FD Fab (**d**) at 37 °C. Note: Black solid lines are fitted curves using a 1:1 binding model, and colored lines are the actual data. Concentrations of recombinant FD (from bottom to top) ranging from 0.02 to 10 nM were run in triplicate. The sensorgrams were generated after in-line reference cell correction followed by buffer sample subtraction.

The goals of the MIC study were to support the IND filing in several ways: (1) To evaluate the binding affinities and kinetics of CCFD9260S with human and cynomolgus monkey FD proteins and (2) to evaluate whether PEG conjugation impacted anti-FD Fab binding to its targets. Since FD is secreted in soluble form, an SPR binding assay was chosen. To compare CCFD9260 and the unconjugated anti-FD Fab binding to FD, it is preferable to keep them in solution as analytes so that the molecules are not modified. In this case, the final PEGylated material was very heterogeneous. The number of conjugated Fab molecules on each PEG ranged from fewer than 6 to greater than 8, with ~60% of the material having 7 or 8 Fab molecules. This requires CCFD9260 and the unconjugated anti-FD to be captured or immobilized on the sensor chip as ligand. The multivalency and the heterogeneity of the PEGylated material would otherwise add challenges to data analysis and prevent accurate determination of kinetics and affinities when injected as the analyte. As mentioned in case study 2, the indirect capture format offers multiple advantages over the direct immobilization format. The requirement of using the indirect format, though, is that the ligand protein must be tightly captured by the capturing antibodies so that it generates a stable baseline for the binding interaction between the ligand and the analyte molecules. In this case, an indirect capture format was first attempted, where the PEGylated anti-FD and unconjugated anti-FD Fab were captured by an anti-human IgG Fab antibody (GE Healthcare) on the sensor chip, and recombinant FD proteins were injected as analytes. As shown in Supplementary Figure S2, surface stability test results revealed that a significant amount of anti-FD dissociated from the anti-Fab capture antibody, indicating that this format is not suitable to capture anti-FD Fab for affinity measurement. A similar observation was also reported before on an indirect capture format where an anti-VEGF Fab was captured by the anti-Fab antibody [3]. Therefore, binding affinities and kinetics of CCFD9260S to FD proteins were determined using an assay format in which the PEGylated anti-FD and unconjugated anti-FD Fab were immobilized on the sensor chip, and recombinant FD proteins were injected as analytes. As shown in Figure 3, CCFD9260S antibody bound to human and cynomolgus monkey FD proteins with high affinities. The K_D_ values determined using a 1:1 binding model for the binding of CCFD9260S to human and cynomolgus monkey FD were 136 and 369 pM, respectively, and the K_D_ values for anti-FD Fab were 168 and 394 pM, respectively. These data verified the Tox species selection, and also proved that PEG conjugation did not affect anti-FD binding to FD targets as CCFD9260S had very similar binding affinity and kinetics compared with the unconjugated anti-FD Fab. 

This case study provides a strategy for assessing the binding interaction of multivalent and heterogeneous antibody fragments to its target antigen, and demonstrated that understanding technical limitations of different assay designs is critical to the development of a successful fit-for-purpose MIC strategy. 

## 5. Case Study 4—MIC Strategy to Characterize Immuno-PET CD8 Antibodies Binding to the Targets

**Figure 4:**
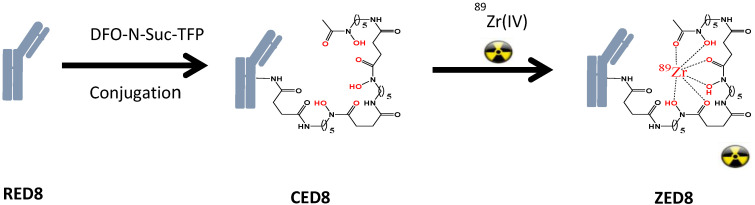
Anti-CD8: the immuno-PET imaging agent. DFO-N-Suc-TFP = N-succinyl-desferrioxamine-tetrafluorphenol ester.

CD8 is a transmembrane glycoprotein characteristic of effector T lymphocytes, that are believed to be central to the anti-tumor activity of cancer immunotherapy (CIT) [55]. Imaging the distribution of CD8−positive T lymphocytes may reveal aspects of the immunobiological status of patients receiving (or eligible to receive) CIT and to help guide CIT development. The clinical lead molecule, CED8 is derived from an one-armed CD8 monovalent antibody (RED8) that is conjugated with N-succinyl-desferrioxamine (DFO), which then becomes the imaging agent by chelation of a trace amount of the radioisotope ^89^Zr (ZED8) [56] (Figure 4). RED8 binds the ECD of CD8 alpha (CD8α). Images of the antibody distribution are acquired using PET, which enables non-invasive monitoring of the distribution of the CD8 antibody after it is administrated to patients.

In order to verify the dose selection in the IND-enabling GLP toxicity study in cynomolgus monkey, it is critical to derive accurate binding affinities of CD8 antibodies to cynomolgus monkey and human CD8 targets. In addition, it is important to evaluate the impact of the DFO conjugation on the binding of CD8 antibody to its targets. Since CD8 is a cell surface protein, and ZED8 itself already has the radioisotope ^89^Zr incorporated, cell-based radiolabeled equilibrium assays were first used to determine binding affinities of ZED8 to CD8 expressed on human and cynomolgus monkey PBMCs. The radiolabeled equilibrium assays, however, were found to be unsuitable for characterizing binding affinities of the anti-CD8 antibody to human and cynomolgus monkey cellular targets. First, the assays suffered from significant non-specific binding that constituted much of the total signal. Second, due to the low binding affinity of the CD8 antibody to cynomolgus monkey CD8, the binding isotherm did not reach a plateau even at the highest tested concentration of ZED8, which indicated that receptors on cells are not saturated (data not shown). As saturation of receptors is required to derive reliable K_D_ values using cell-based equilibrium assay, an alternative SPR binding assay that can accurately compare the binding affinities of the CD8 antibodies to human and cynomolgus monkey CD8 proteins was needed.

In the SPR assay, the binding kinetics and affinities of the CD8 antibodies to recombinant CD8α ECD proteins were first evaluated using an assay format (format 1) in which the recombinant human and cynomolgus monkey CD8α ECD proteins were immobilized on a sensor chip, and the CD8 antibodies were injected as analytes. This format was preferred because CD8 is expressed on the surface of cells and the available recombinant CD8α ECD Fc-fusion proteins contained two binding domains. Thus, the binding interaction would be a simple 1:1 binding. However, it was found that the data could not be described well by a 1:1 binding model (data not shown), indicating that there were multiple populations involved in the binding. This is possibly caused by heterogeneous population of CD8α ECD proteins after being immobilized on the sensor chip via amine coupling chemistry. Thus, an alternative assay format (format 2) was explored where the CD8 antibodies were immobilized on a sensor chip and CD8α ECD proteins were injected as analytes. Since antibodies are well-known to have excellent stability properties, they might be more consistent in their orientation after being immobilized on the sensor chip. A potential issue of using this format is that bivalent binding interactions could happen because of the bivalent nature of the recombinant CD8α ECD fusion proteins, which could cause the determined affinity to appear higher [57]. To circumvent this issue, very low densities of CD8 antibodies (R_max_ less than 5 RU) were immobilized on the sensor chip to encourage monovalent interaction. 

**Figure 5:**
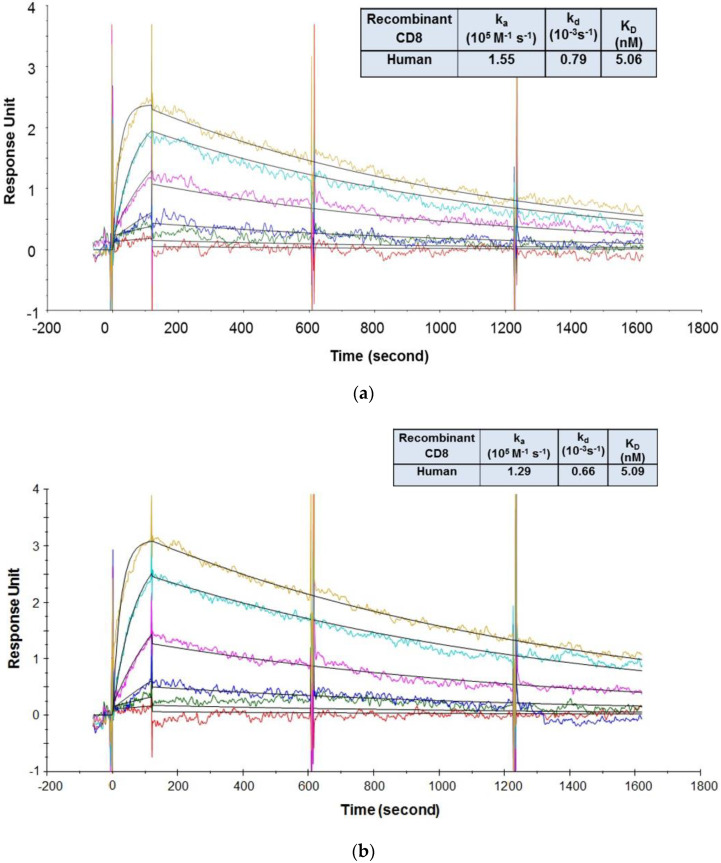

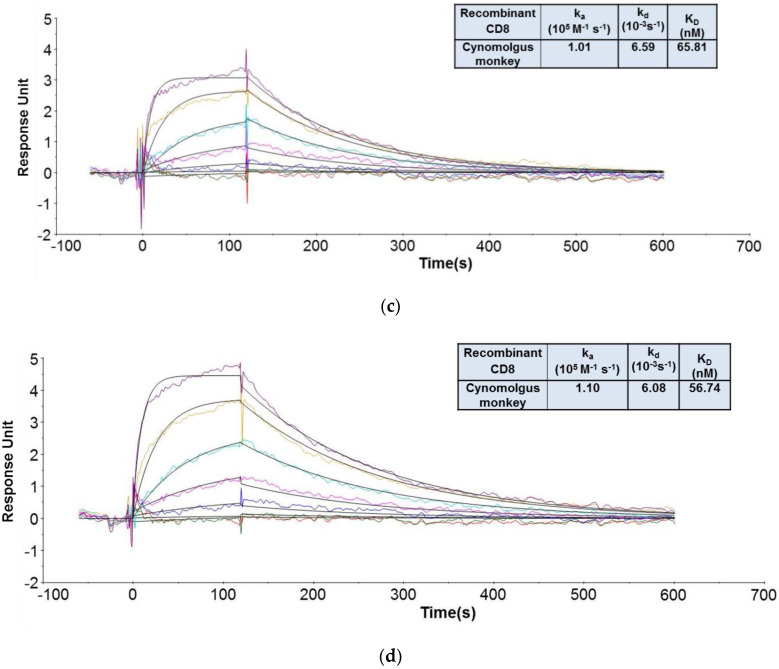
Representative sensorgrams of concentration-dependent binding of the recombinant human CD8 to CED8 [56] (**a**) and RED8 (**b**), and recombinant cynomolgus monkey CD8 to CED8 (**c**) and RED8 (**d**) at 37 °C. Note: Black solid lines are fitted curves using a 1:1 binding model and colored lines are the actual data. Concentrations of the recombinant human CD8 protein (from bottom to top in the upper panel) are 1.23, 3.70, 11.1, 33.3, 100, and 300 nM. Concentrations of the recombinant cynomolgus monkey CD8 protein (from bottom to top in the upper panel) are 1.23, 3.70, 11.1, 33.3, 100, 300, and 900 nM. Sensorgrams were generated after in-line reference cell correction followed by buffer sample subtraction. The kinetics and affinity data in the table in 5a, 5b, 5c, and 5d were averaged from 3, 2, 6, and 5 independent experimental SPR runs, respectively.

As shown in Figure 5, the recombinant human CD8α ECD bound to CED8 and RED8 with average K_D_ values of 5.06 [56] and 5.09 nM, respectively. The recombinant cynomolgus monkey CD8α ECD bound to the CED8 and RED8 with average K_D_ values of 65.81 and 56.74 nM, respectively. No significant or systematic discrepancy was observed between the experimental data and the fitted curves, suggesting that the 1:1 binding model adequately described the interactions. The CD8 antibody to be evaluated in the cynomolgus monkey GLP study, CED8, bound to the recombinant human CD8α ECD fusion protein with an affinity 13-fold higher than that to the recombinant cynomolgus monkey CD8α ECD fusion protein, a difference attributed to a slower dissociation rate. The data supported the dose selection for the IND-enabling cynomolgus monkey GLP toxicity study, and also demonstrated that the DFO conjugation did not have an impact on the CD8 antibody binding to its targets.

This study highlighted the importance of designing fit-for-purpose MIC assays so that the derived affinity data addresses specific needs in support of toxicity studies during development. 

## 6. Conclusions

Fit-for-purpose MIC assay strategies are critical during early development of therapeutic antibodies to enable key decisions. The successful design of the MIC assay strategy for a program rely on many factors, including target biology, biophysical properties of the therapeutics, mechanism of interaction, relevant binding species, as well as logistical considerations such as accessibility of cell lines, supply of assay reagents, timeline, and resources. As affinity measurement is context-dependent, it is critical to choose the assay design (assay format, conditions, etc.,) that is most relevant to the question(s) to be addressed. Therefore, a sound MIC strategy that is based on the thorough understanding of the needs and the technical feasibility should be developed prior to in-depth technical work. This outcome is best achieved through discussions with team members from different functions such as pharmacology, toxicology, biology, and manufacturing. In addition, because MIC assay results are dependent on reagents used, it is highly desirable to assess the quality of critical reagents right before they are used in the MIC assays. This practice is especially important for reagents that are prone to aggregate, have gone through multiple freeze-thaw cycles, or are generated for the first time with unknown stabilities. It is worth pointing out that reagents that work in other types of assays do not necessarily possess the quality required for MIC assays. Therefore, besides characterizing MIC assay reagents by traditional analytical tools such as SDS-PAGE, SEC-MALS, and mass spectrometry, it is highly desirable to evaluate binding activity of proteins when conducting MIC assays with appropriate assay format.

The introduction of new antibody modalities provides various advantages over conventional antibodies, meanwhile poses unique challenges to the development of MIC assays primarily due to their complex structural and molecular properties. Compared to conventional mAbs, traditional strategies involving single MIC assay development is unlikely sufficient to address the development need of these programs any more. This may require MIC strategies that apply a diversity of traditional and unconventional approaches to overcome challenges that are associated with these new modalities and to address their specific needs. With additional new antibody-related modalities in development covering a wide range of diversified platforms [20,58,59], this principle should also be applicable to MIC assay development of other new modalities. Thus it is important to work closely with the PK scientist and the toxicologist for each program to understand the development need, and then design MIC assay strategies on a case-by-case basis. We believe the knowledge and experience presented here will help to increase the awareness of the importance of MIC studies in support of therapeutic antibody development and thereby better shape best practices within the community.

## Figures and Tables

**Table 1:** Binding affinity determined by radiolabeled equilibrium assays using different cell lines.

	Human	Cynomolgus Monkey	Rat
Unconjugated lead MSLN antibody	0.2–1 nM ^a^	No binding	No binding
Unconjugated surrogate MSLN antibody	Not applicable	1.3–6.4 nM ^b^	2.7–7.3 nM ^c^

^a^ Analyzed in human BJAB, 293, and HT1080 [47] cell lines expressing MSLN; ^b^ Analyzed in cynomolgus monkey BJAB, 293, and HT1080 cell lines expressing MSLN; ^c^ Analyzed in rat BJAB, 293, and 4/4-RM4 cell lines expressing MSLN.

**Table 2:** Binding kinetics and affinities of mesothelin (MSLN) antibodies to immobilized recombinant human and Cynomolgus monkey MSLN extracellular domain (ECD) proteins determined by Biacore.

MSLN Antibody	MSLN ECD *	K_a1_	K_d1_	K_D1_
		(10^6^M^−1^·s^−1^)	(10^−3^s^−1^)	(nM)
Lead (ADC)	Human	1.8	2.5	1.4
Lead (unconjugated)	Human	1.1	2.5	2.3
Surrogate (ADC)	Cyno	0.3	2.5	7.9
Surrogate (unconjugated)	Cyno	0.2	2.8	11.9

* Expressed by CHO cells (the least glycosylated fraction). Binding kinetics and affinity data were calculated with BIA evaluation Software (version 3.2) (GE Healthcare; Piscataway, NJ, USA) using a bivalent analyte binding model. k_a1_ = first association rate constant; k_d1_ = first dissociation rate constant; K_D1_ = first equilibrium dissociation constant. The data were averaged from three independent experimental runs. Experiments were conducted at 37 °C.

**Table 3:** Characterization of 1G7 binding to FcRH5- and CD3-expressing cells.

	K_D_ (nM)	C_half_ (nM)
Human FcRH5	3.1 ^a^ [50]	N/A
Cynomolgus Monkey FcRH5	8.2 ^b^ [50]	N/A
Human CD3	2.6 ^c^	4.2 ^d^
Cynomolgus Monkey CD3	N/A	2.2 ^e^

^a^ Analyzed in human SVT2 cell lines expressing FcRH5 at room temperature; ^b^ analyzed in cynomolgus monkey SVT2 cell lines expressing FcRH5 at room temperature; ^c^ analyzed in human Jurkat cell lines expressing CD3 at room temperature; ^d^ analyzed in human PBMC cells expressing CD3 at 4 °C; ^e^ analyzed in cynomolgus monkey PBMC cells expressing CD3 at 4 °C. K_D_ = equilibrium dissociation constant; C_half_ = concentration at half maximal response. The data were averaged from three independent experimental runs.

## Supplementary Materials

The following are available online at https://www.mdpi.com/2073-4468/9/2/7/s1, Figure S1: Ratio of Rmax (E) and Rmax (T) of recombinant MSLN ECD expressed by (**A**) E.coli; and (**B**) CHO cells (fractions with the least glycosylation). Rat MSLN ECD expressed by CHO cells was not tested due to unavailability of the protein. Rmax (E) and Rmax (T) are maximum binding responses determined experimentally and theoretically, Figure S2: Surface stability test of anti-FD Fab. Level of anti-FD Fab captured by an anti-Fab antibody over 15 minutes in an indirect capturing format at 37 °C. Level of anti-Fab antibody immobilized was approximately 8000 RU, and anti-FD Fab was indirectly captured at approximately 80 RU.
