# Supplementary material for: Molecular Interaction Characterization Strategies for the Development of New Biotherapeutic Antibody Modalities
Source: Antibodies (Basel). 2020 Mar 25;9(2):7. doi: 10.3390/antib9020007 (PMC7344756; doi:10.3390/antib9020007)
Supplement: Supplementary file 1 [file antibodies-09-00007-s001.pdf]

Supplementary Figure 1

1A

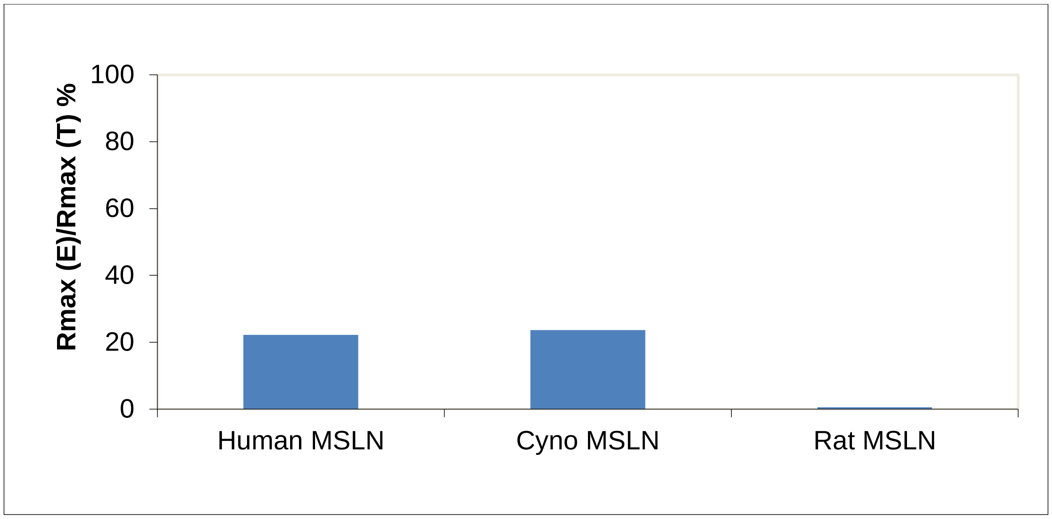

1B

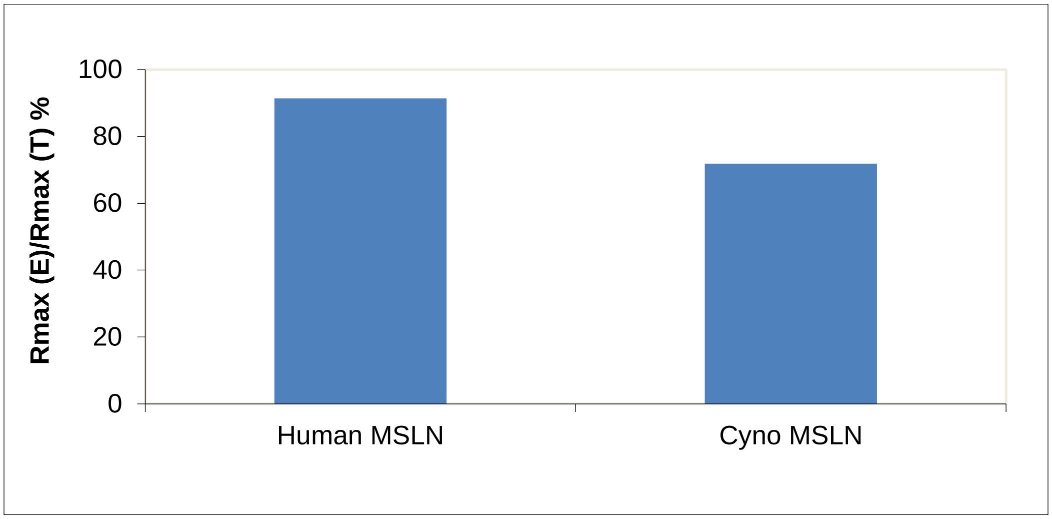

Supplementary Figure 1. Ratio of Rmax (E) and Rmax (T) of recombinant MSLN ECD expressed by (A) E.coli; and (B) CHO cells (fractions with the least glycosylation). Rat MSLN ECD expressed by CHO cells was not tested due to unavailability of the protein. Rmax (E) and Rmax (T) are maximum binding responses determined experimentally and theoretically.

Supplementary Figure 2.

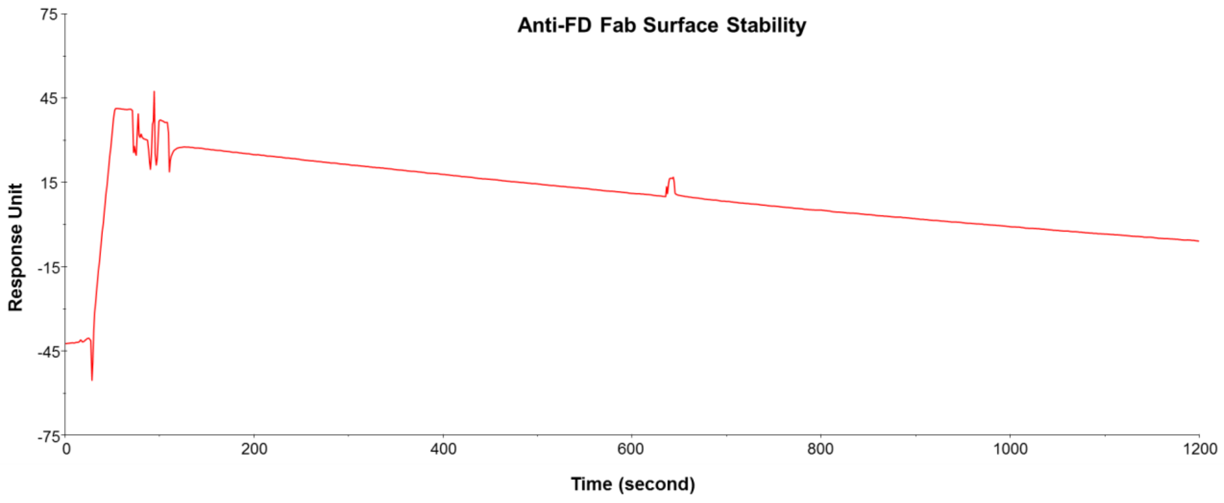

Supplementary Figure 2. Surface stability test of anti-FD Fab

Level of anti-FD Fab captured by an anti-Fab antibody over 15 minutes in an indirect capturing format at 37 °C. Level of anti-Fab antibody immobilized was approximately 8000 RU, and anti-FD Fab was indirectly captured at approximately 80 RU.
